# Microneedles: Characteristics, Materials, Production Methods and Commercial Development

**DOI:** 10.3390/mi11110961

**Published:** 2020-10-27

**Authors:** Amina Tucak, Merima Sirbubalo, Lamija Hindija, Ognjenka Rahić, Jasmina Hadžiabdić, Kenan Muhamedagić, Ahmet Čekić, Edina Vranić

**Affiliations:** 1 Department of Pharmaceutical Technology, Faculty of Pharmacy, University of Sarajevo, Zmaja od Bosne 8, 71000 Sarajevo, Bosnia and Herzegovina; merima.sirbubalo@ffsa.unsa.ba (M.S.); lamija.hindija@ffsa.unsa.ba (L.H.); ognjenka.rahic@ffsa.unsa.ba (O.R.); jasmina.hadziabdic@ffsa.unsa.ba (J.H.); 2 Department of Machinery Production Engineering, Faculty of Mechanical Engineering, University of Sarajevo, Vilsonovo šetalište 9, 71000 Sarajevo, Bosnia and Herzegovina; kenan.muhamedagic@mef.unsa.ba (K.M.); cekic@mef.unsa.ba (A.Č.)

**Keywords:** transdermal drug delivery, microneedles, microneedle arrays, materials, microscale fabrication techniques, coating techniques

## Abstract

Although transdermal drug delivery systems (DDS) offer numerous benefits for patients, including the avoidance of both gastric irritation and first-pass metabolism effect, as well as improved patient compliance, only a limited number of active pharmaceutical ingredients (APIs) can be delivered accordingly. Microneedles (MNs) represent one of the most promising concepts for effective transdermal drug delivery that penetrate the protective skin barrier in a minimally invasive and painless manner. The first MNs were produced in the 90s, and since then, this field has been continually evolving. Therefore, different manufacturing methods, not only for MNs but also MN molds, are introduced, which allows for the cost-effective production of MNs for drug and vaccine delivery and even diagnostic/monitoring purposes. The focus of this review is to give a brief overview of MN characteristics, material composition, as well as the production and commercial development of MN-based systems.

## 1. Introduction

The application of various chemical agents on the skin dates back thousands of years, when they were applied to treat diseases, protect the skin, or for cosmetic reasons [1]. The ancient Greeks made a balm from a mixture of water, olive oil, and lead (II) oxide, whereby olive oil and lead (II) oxide had an occlusive and astringent effect, respectively [2]. However, the skin was considered as an impermeable membrane until 1893, when Bourget proved that the topical application of salicylic acid could treat acute rheumatoid arthritis [3,4]. 

At the beginning of the 20th century, lipophilic agents were discovered to increase skin permeability, and using Wolf’s tape stripping technique, Blank concluded that the *stratum corneum* (SC) represents the main barrier for the penetration and permeation of active pharmaceutical ingredients (APIs) [5,6]. 

Skin, as a drug delivery route to the systemic circulation, was neither commercially nor scientifically employed until 1954, when it was shown that 2% nitroglycerin ointment could control angina pectoris. Therefore, this ointment was the first commercial preparation formulated for the transdermal delivery of API into the systemic circulation [4,7].

## 2. Transdermal Drug Delivery

Oral administration represents the most common delivery route for the majority of drugs. However, pH changes in the body that cause drug degradation, enzymatic activity, or first-pass metabolism effect are the main problems linked to this drug delivery route [8].

Drugs that cannot be taken orally are usually given by hypodermal injections. However, this way of administration may cause patients fear, pain, infection, or skin injury [9], and requires trained staff for drug application [10,11,12,13,14]. As an alternative strategy, APIs can be administered transdermally in the form of gels, creams, ointments, or patches [8]. This approach allows for controlled drug release in a minimally invasive manner compared to the bolus drug delivery via hypodermic needles [15].

The transdermal route is used to slowly transport API molecules from the surface of the skin into the body, overcoming problems associated with the oral delivery [8,10]. This way is generally well accepted by patients since, for some indications, it is more convenient and efficient to “put on a patch“ than to “pop a pill“ [16]. Although the transdermal patch market was worth $6.23 billion in 2019 [17], the biggest problem is the limited number of APIs (less than 20) that are suitable for transdermal delivery [8,18]. Furthermore, API penetration rate depends on age, race, site of application, and skin condition [19].

This administration route is the most appropriate for APIs with a low molecular mass (less than 400–500 Da), balanced lipophilicity (log *p* 1–3), and low melting point [20,21]. Besides, a high pharmacological potential of APIs and a dose of a few milligrams that achieves a therapeutic response are prerequisites [22]. Drugs with log *p* values < 1 cannot penetrate through the SC efficiently, because they are too hydrophilic. On the other hand, drugs with log *p* values > 3 are highly hydrophobic and they become trapped in intercellular lipids of the SC shortly after topical administration [18].

Although the blood circulation is close to the skin surface, the delivery of hydrophilic drugs, charged molecules, peptides, proteins, and nucleic acids represents a challenge, since the skin is adapted to prevent the entry of foreign substances [15,23]. The complexity of the drug penetration process into the skin depends both on the lipid matrix structure and mechanical properties of the skin, i.e., its parts that are influenced by environmental conditions (humidity and temperature) [24].

To overcome the barrier function of the SC, achieve controlled drug release, and improve the effectiveness of existing formulations for transdermal administration, intensive research on physical and chemical methods for therapeutic drug delivery has been carried out. These methods include iontophoresis, which drives hydrophilic drugs through sweat glands and hair follicles, as well as electroporation, sonophoresis, and laser or chemical enhancers that create nanometer-scale disturbances in the SC for drug molecules passage [18,25,26,27]. Nevertheless, it is still a significant challenge to deliver substances such as macromolecules into the skin [25].

Microneedles (MNs) enable a transport path for small drug molecules, macromolecules, or nanoparticles [28] and deliver APIs that cannot passively pass through the SC. Importantly, the size of the drug molecule is not a limiting factor for their application [13,29].

Therefore, this review aims to summarize the current knowledge of MN classification, methods, and materials used in their manufacture, and provide examples of the applications of this technology.

## 3. Microneedles—Classification and History

The concept of miniature needles for drug delivery came about in the 1960s, and it was patented by Alza Corporation in 1971. That device contained tiny protrusions (MNs) and a drug reservoir for API delivery by diffusion or pressure. This MNs concept was experimentally tested almost 30 years later, with the extensive development of microfabrication manufacturing technology. From the first published scientific paper on drug delivery via MNs [30], the interest in MNs has grown in scientific circles, along with the number of clinical trials and approved products based on this principle [31].

Briefly, MNs represent solid or hollow tubes with a length of 50–900 μm, less than 300 μm in diameter, of various shapes, sizes, and densities per unit area of supporting or adhesive membrane surface [32,33]. Arrays of a maximum of 20,000 MNs per cm^2^ on the base plate create a physical path of micron dimensions through the upper layers of the epidermis, without reaching the nerve endings (nociceptors) in the dermis.

**Figure 1:**
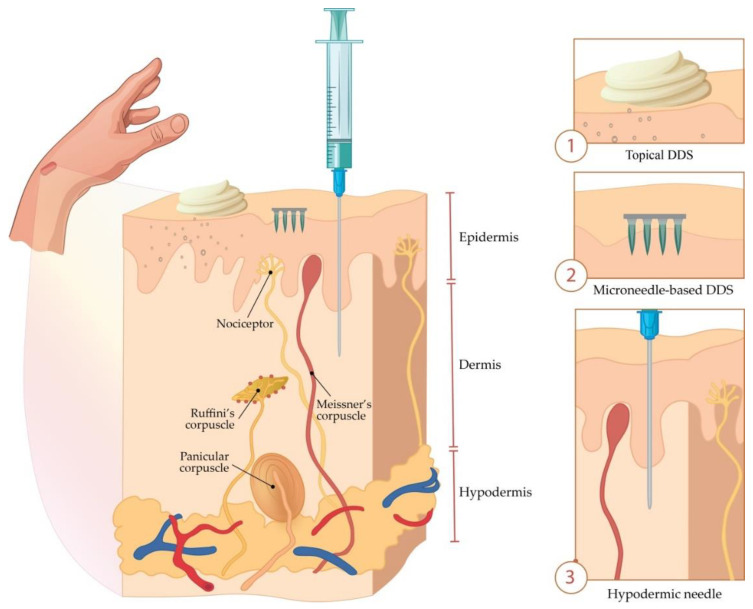
Comparison of drug delivery systems (DDS) based on (1) the conventional topical formulation, (2) microneedles (MNs), and (3) hypodermal injection.

A comparative view of conventional topical drug delivery system (DDS), MNs, and hypodermal injection is shown in Figure 1.

### 3.1. Classification of Microneedles

**Figure 2:**
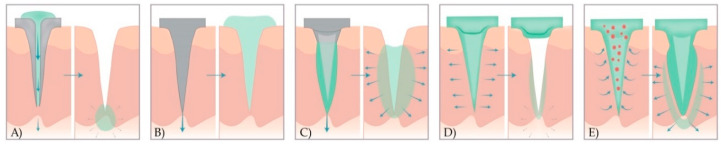
A schematic representation of drug release methods with five different types of MNs. (**A**) Hollow MNs pierce the skin (left) and provide the release of liquid drug formulation through the needle lumen (right). (**B**) Solid MNs pretreat the skin to create transient microchannels skin (left) and increase the permeability of the drug that is then, applied in the form of a transdermal patch, solution, cream, or gel (right). (**C**) Coated MNs with drug formulation (left) enable the fast dissolution of the coated drug in the skin (right). (**D**) Dissolving MNs are prepared from polymer and embedded drug in the MN matrix (left) to provide the bolus or controlled delivery of a drug (right). (**E**) Hydrogel MNs poke the skin, uptake interstitial fluids (right), and induce diffusion of the drug from the patch through the swollen MNs (left).

There are several ways to classify MNs. Some authors divide them into solid and hollow MNs and include coated, uncoated, and dissolving MNs in the category of solid ones [34]. Others divide them according to the production method into “in-plane MNs” (microneedle shafts oriented parallel to the base substrate) and “out-of-plane MNs” (microneedle shafts bent at 90° to the base substrate) [35]. The most common categorisation is into four (five) types (Figure 2) [36]:

- *hollow MNs*—used for the injection of liquid drug formulations through the MN bores into the skin;
- *solid MNs*—used for the pretreatment of skin before administration of APIs from the external reservoir;
- *coated solid MNs*—used for the continuous dissolution of APIs in the skin, as the drug is coated on the MN shaft and tips;
- *dissolving MNs*—that dissolve completely in the skin and thus release drugs or vaccine incorporated into the MN matrix; and
- *hydrogel MNs*—that swell up upon administration and API release from the patch through swollen MNs.

#### 3.1.1. Hollow Microneedles

In contrast to solid MNs, hollow MNs possess a lumen or internal bore (5–70 μm wide), and thus enable the transportation of the drug [37] by passive diffusion or by applying pressure using a syringe, pump, or gas [38,39,40,41]. Liquid formulation flows (typically, 10–100 μL/min) from the reservoir through hollow MNs and continuously release its content in certain layers of the skin (poke and flow) (Figure 2A) [21,37,42,43]. Commonly, they are used to deliver insulin [44,45] and vaccines [28].

In comparison to other MN types, hollow ones deliver higher API amounts. The ease of their manufacturing, low costs, and accurate drug release control from the liquid preparation are the advantages of this MN type [46]. Furthermore, controlled API release can be achieved by the incorporation of a microfluidic chip [47,48] or micropump [49] into an array of MNs [35,50]. The integration of the drug reservoir with a heater delivers the drug solution to the skin by spreading the liquid or creating bubbles. By pressing the flexible reservoir, the drug solution is released into the skin [34,46,50]. Although this therapeutic strategy requires the liquid drug formulation, no reformulation is necessary. However, a challenge is to deliver a dry formulation, generally used to improve drug stability and convenience of patch-based application, without reconstitution [31]. The main limitations are the possibility of clogging the needle tip in the tissue and the resistance to flow due to the density of compressed skin tissue around the MN tip. Therefore, to overcome this disadvantage, a side opening with off-centred holes is designed [37]. Another way is the gradual insertion of the needle [18].

#### 3.1.2. Solid Microneedles

Solid MNs can be applied both with or without drug coating, in the poke and patch approach. In the first case, MNs are initially applied to the skin to create transient aqueous microchannels in the SC. After the removal of MN array, the conventional drug formulation in the form of transdermal patch, solution, cream, or gel is applied to that region (Figure 2B) [42,43]. Therefore, API from the external reservoir permeates through these microchannels by passive diffusion [18].

In the second case, solid MNs are not only used for piercing the skin but also serve as drug reservoirs when MNs are coated with the suitable drug formulation. Although this approach allows for the rapid delivery of the therapeutic API dose, a limited drug amount can be applied on the base and shaft of the MN (usually less than 1 mg) [51]. Furthermore, the integration of these MNs, as a part of a closed-loop system of a smart patch allows for the controlled drug delivery based on feedback from the analysis of body fluids [52,53]. With these systems, the incorporation of lab-on-a-chip technology will enable the detection of broadly employed biomarkers not only in hospitals but in clinical laboratories as well [53].

#### 3.1.3. Coated Microneedles

Coated MNs are manufactured by coating solid MNs with a drug formulation before application on the skin (Figure 2C). Once the coated arrays of MNs are inserted into the skin, the drug formulation is continuously dissolved and then released into the skin (coat and poke approach). Coated MNs rapidly deliver macromolecules such as vaccines [54,55,56,57], proteins [58], peptides [59,60,61], and deoxyribonucleic acid (DNA) [62]. Although this type of MN enables a simple one-step application process, the main drawback is the limited amount of drug that can be coated on the surface of the MNs. The use of coated MNs is, therefore, limited to potent molecules or drugs [18].

#### 3.1.4. Dissolving Microneedles

Besides APIs, dissolving MNs consist of soluble matrix containing biocompatible polymers or sugars. MN tips dissolve after contact with the interstitial fluid, followed by API release (poke and release approach) (Figure 2D). As the release kinetics of the active substance depend on the degree of dissolution of the constituent polymers, it is possible to control drug delivery by adjusting the polymer composition or by modifying the manufacturing process [18]. Ling and Chen presented a dissolving MN patch consisting of starch, gelatin and insulin as a drug model. An in vitro test showed that these MNs released almost all of the insulin content within 5 minutes. Furthermore, they were mechanically strong enough, allowing bioactive molecules to be stably encapsulated [63].

Nowadays, there is growing interest in dissolving MNs made of biodegradable materials, as they enable API delivery without creating sharp, bio-contaminated, and non-degradable waste [31]. Moreover, in the case of the production of MNs from semi-synthetic and synthetic polymers and sugars, the manufacturing costs are significantly lower [64]. However, the main disadvantage is the deposition of polymers in the skin, which is undesirable for long-term use [18]. Degradable MNs, a subcategory of dissolving MNs, may deliver a wide range of hydrophilic agents, including caffeine, lidocaine, metronidazole, ibuprofen, as well as several biopharmaceutical molecules (low molecular weight heparin, insulin, leuprolide acetate, erythropoietin, and human growth hormone) [65].

#### 3.1.5. Hydrogel Microneedles

A relatively new type of MNs is fabricated from a hydrogel-forming matrix and was first described in 2010 [66]. This new strategy includes integrated systems consisting of cross-linked polymer micro protruding from a solid patch-like base containing the API. Upon administration, needles rapidly uptake interstitial fluid from the tissue, inducing the diffusion of the drug from the patch through swollen MNs (Figure 2E). These systems are manufactured from aqueous mixtures of specific polymeric materials, i.e., polymethylvinylether-co-maleic acid (PMVE/MA) [18]. Lee et al. made hydrogel MNs from ultra-low viscosity carboxymethylcellulose (CMC) and amylopectin. After the incorporation of sulforhodamine, bovine serum albumin, or lysozyme, MNs were dissolved safely in the skin, therefore allowing bolus or sustained release delivery [67]. Garland et al. showed that drug delivery could be adjusted by modulating the density of the hydrogel matrix. Importantly, after the removal of the hydrogel MNs, the skin was intact, leaving no polymer residues. Hydrogel MNs have softened enough to prevent re-application, thus reducing the risk of transmitting infections [68].

Phase transition MNs are a subcategory of hydrogel MNs. After the absorption of the body fluid, the API from the MN matrix is released due to swelling of the polymer. This MN type leaves some or no residue after application [36]. Although dissolving or degradable MNs could carry higher concentrations of the drug, the matrix makes up a large portion of the needles, which dissolve or degrade in the skin, making them unsuitable for everyday use. Retaining the matrix in the skin may cause low compliance in patients and potential side effects if the drugs are administered daily or at short intervals. The most suitable active ingredients, in this case, are vaccines because they are used only once (or a few times), so the amount of matrix deposited in the skin will be more acceptable. Therefore, the most promising type of MN is one made of hydrogel that does not dissolve nor degrade in the skin but has a controlled or continuous release of active substances [36].

## 4. Microneedle Production

### 4.1. Materials

The advent of microfabrication manufacturing technology in recent decades has enabled the development of MNs in research laboratories and pharmaceutical companies [69]. Therefore, it is necessary to select the most suitable materials for MN production based on the following criteria [70]:

- gentle manufacturing without damaging sensitive and unstable molecules;
- controlled or immediate drug release; and
- sufficient mechanical strength for skin penetration.

The first solid MNs were made of silicon [30], as industrial high-precision microelectronics tools and silicone flexibility enabled the production of MNs. However, their main disadvantage is the breakage of the silicon MN due to their brittle nature. Nowadays, MNs come in a variety of shapes and sizes, as well as materials (Table 1), including stainless steel [22,71,72], titanium, nickel-iron, glass [40,44], and ceramics [73]. Metal MNs have sufficient mechanical strength to penetrate the skin, but their disadvantage is that they generate potential biological waste [8,10]. Interestingly, nitinol is used in vascular surgery due to its advantages in terms of elasticity, shape-memory capability, and biocompatibility [74]. However, polymeric MNs have better solubility and usage in case of the tip breaking [75]. Water-soluble polymers [10,76,77,78] and engineering plastics such as CMC, poly (glycolic acid) (PGA), polylactic-co-glycolic acid (PLGA), poly (vinyl alcohol) (PVA), poly (vinylpyrrolidone) (PVP), polylactic acid (PLA), chondroitin sulfate, and polycarbonate are employed for MN production, whereas dissolving MNs are composed of sugars such as maltose [79,80], dextran [81], or galactose [31,82,83,84]. The suitable materials for MN production are summarized in Table 1.

The selected material determines the manufacturing MN method that should be accurate, reproducible, robust, and precise [107]. The manufacturing methods for solid or hollow MNs, described in the following sections, include MEMS, lithography methods, laser cutting, laser ablation, metal electroplating, isotropic and anisotropic etching [92], injection molding [108], DAB method [109], surface/bulk micromachining, polysilicon micromolding [110], and additive manufacturing (AM) technologies (FDM [111], stereolithography (SLA) [47,112,113], digital light processing (DLP), and 2PP [114]). Additionally, the coating of MNs with a formulation that contains APIs is described in detail below.

### 4.2. Microneedle Production Methods

#### 4.2.1. Microelectromechanical Systems (MEMS)

**Figure 3:**
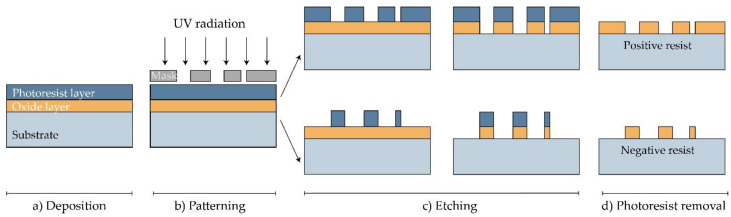
Manufacturing of MNs using photolithography [117]. (**a**) Deposition: As a substrate, Si wafer is exposed to steam or humidified to produce the wafer with an oxide coating. Then, the photoresistive material is spin-coated onto a substrate. (**b**) Patterning: Mask guided UV radiation is exposed to the photoresistive material. (**c**) Etching: soluble resist material is removed and SiO_2_ film etched. (**d**) Photoresist removal: in this step, the photoresist layer is removed.

Solid and hollow MNs, as well as molds for dissolving MNs, have been manufactured directly from a suitable material substrate using MEMS methods [31]. The production involves a precisely controlled three-step process: deposition, patterning, and etching of materials (Figure 3) [70,115]. Complex three-dimensional (3D) structures are, therefore, formed due to differences in the selectivity to the etchant between different materials [115].

In the first step, a film with a thickness between a few nanometers and 100 μm is formed on a substrate by a chemical (CVD) or physical vapor deposition (PVD) [70,116,117]. In the PVD process, the film is formed by atoms transferred directly from the source to the substrate through the gas phase. In the CVD process, the chemical reaction on the substrate surface results in film formation [117].

Then, a two-dimensional master pattern of the desired material is transferred from the original photomask to the photosensitive-coated substrate during the second phase of the process, called patterning. In most cases, a silicon wafer is used as a substrate, and the transferring process is made using a radiation source with one of the lithography process (photolithography [82], ion beam lithography, or X-ray lithography [77]) [118].

The most common type of lithography is photolithography, a process based on the fact that some materials such as metals are not transparent when exposed to UV light (*λ* = 193–236 nm), while others such as glass are transparent. In this process, an optic mask, an opaque template for generating the desired pattern in a wafer, is created (Figure 3). The mask, which consists of a quartz plate or flat glass, allows light to pass only throughout a defined pattern [119]. The silicon substrate is first exposed to steam or humidified oxygen at about 900 °C to produce an oxide layer, and then, rotated and coated with an organic polymer sensitive to UV light, the so-called photoresist material [18,117,118]. The heat of 75–100 °C followed by UV radiation removes the solvent and forms the desired photo-resistant pattern [119]. In this step, two types of resist, positive and negative, can be used. In the positive resist, the chains of the photo-resistant polymer break up after exposure to UV light, making them more soluble in the chemical solution—the developer, in comparison to the negative resist, where the chemical bonds are strengthened (Figure 3) [18].

Photolithography also enables manufacturing molds for MNs. In this case, a rigid silicone mold with a positive image is made, and then, a negative mold from poly (dimethylsiloxane) (PDMS) is followed by the application of the chosen material [117].

The etching is achieved by applying a strong acid or caustic agent to etch out the uncovered parts of the substrate to form a design on the surface of the material. Two types of etching can be distinguished: wet and dry etching [70]. In the wet etching process, to produce metallic or silicon MN arrays, an excess of material is removed by submerging the substrate in the chemical liquid. The etching can be performed at the same (isotropic etching) or different rates (anisotropic etching) [70,83].

On the other hand, the dry etching process is achieved by using a vapor phase or plasma etcher. Two main types of dry etching are distinguished: reactive ion etching (RIE) and ion-beam milling (IBM). In the RIE process, the gas excitation into a reactive state enables a reaction between the gas and the substrate. The number of ions that influence the degree of isotropy can be adjusted by controlling the gas pressure. The electric field can accelerate ions and further increase the direction of etching. In the case of the IBM process, inert ions are speeded up from a source to physically remove the material to be etched [18]. Although RIE creates structures, the etching rate is low and it is challenging to maintain a high width-to-height ratio. Deep reactive ion etching (DRIE), often called the Bosch process, is suitable for the production of off-plane MNs. This method is used to produce hollow MNs with a lumen of several hundred micrometres (width to height ratio of 30:1) [84]. Although wet etching can reduce fabrication costs compared to dry etching, the best results are achieved by combining isotropic dry and anisotropic wet etching to produce well-defined and sharp MN tips [31,84,120].

Henry et al. used DRIE to produce silicone, out-of-plane MNs. First, the chromium masking material was applied to a silicon wafer and formed into dots of a diameter equal to the base of the desired MNs. Subsequently, the wafers are exposed to RIE, so that the regions protected by the metal mask are preserved to allow the MNs to form [30]. Wang et al. described the production process of hollow MNs in two steps: firstly, they produced PDMS mold using photolithography to obtain the pyramidal top profile; secondly, they made hollow SU-8 MNs on a built-in PDMS mold. The MNs were heated to 60 °C to increase encapsulation in the micro-trenches by decreasing its viscosity [91].

Paik et al. fabricated an in-plane single, crystal-silicon MN array integrated with PDMS microfluid chip for blood extraction in point-of-care devices and DDS. The MNs were produced by anisotropic dry etching, isotropic dry etching, and the trench-refilling process. They concluded that the MNs are stiff enough to penetrate animal skin models without creating damage [35]. Ma et al. fabricated hollow MNs on the silicon substrate by inductively coupled plasma (ICP) and anisotropic wet etching methods. Subsequently, MNs were integrated with the PTZ pump for precise insulin dosage. They founded that this system can be used for programmable drug delivery or fluid sampling [92].

#### 4.2.2. Laser Cutting

Metal MNs can be manufactured by 3D laser cutting [22,25,51,72,93,94], laser ablation [95,96,97], and electroplating or electroless plating of metal onto positive or negative MN molds [31].

**Figure 4:**
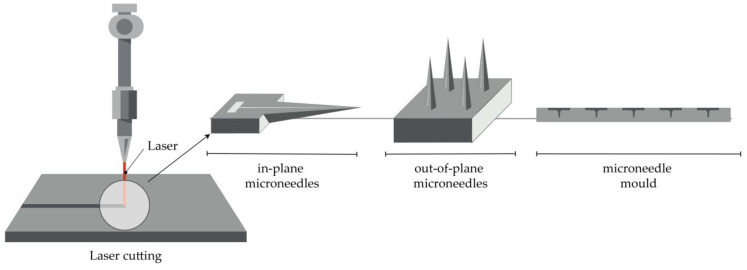
Principle of manufacturing of MNs (in-plane and out-of-plane) and MN molds by the laser cutting.

Arrays of solid MNs are produced by cutting stainless steel or titanium sheets in the shape of MNs with an infrared laser (Figure 4). The desired shape, geometry, and dimensions of MNs are created using some of the computer-aided design (CAD) software. The laser beam follows the predetermined shape of the needle, then MNs are cleaned in hot water and bent at 90 degrees, vertically from the plane of the base. In order to deburr, reduce the thickness of MNs and sharpen the tips, MNs are subsequently electropolished, washed, and dried with compressed air. This manufacturing method can be used to produce a single row of MNs of different geometries, as well as two-dimensional rows of metallic MNs [22,25,51,72,93,94].

Furthermore, the production of hollow MNs [105] and molds for a dissolving MN patch is reported [121]. In the first case, a KrF laser (*λ* = 248 nm) was used to make holes from the side of PLA sheets, which had previously been manufactured using a micromolding technique [105].

Albarahmieh et al. reported on the fabrication of MN patches using a CO_2_ laser on polymethylmethacrylate (PMMA) sheets. Subsequently, a selected mixture was poured into PMMA molds and then, dissolving MNs, containing methylhydroxy-4-benzoate and terbinafine hydrochloride were obtained [121].

#### 4.2.3. Laser Ablation

**Figure 5:**
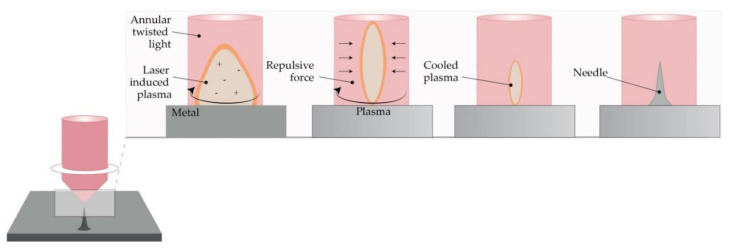
The principle of metal MN fabrication using twisted light with a spin by Omatsu et al. (Modified from [96]).

This method is a top-down method for processing materials, including metals. Light pulses give the bulge of the desired shape on a metal plate, thus forming solid metal arrays [95]. However, due to the high-intensity laser pulses, the formation of plasma of ions and electrons is not suitable for the fabrication of structured materials. Omatsu, therefore, introduced a novel, time and cost-effective fabrication method of manufacturing metal MNs based on circularly polarized optical vortices that have nonzero total angular momentum, as shown in Figure 5. The authors reported on the fabrication of a tantalum MNs with a vertical height of over 10 µm and significantly small tip radii [96].

In 2020, Evens et al. introduced a novel method for the production of solid polymer MNs using laser-ablated steel molds. This mold was also employed in the injection molding process for the production of the polymer MNs. In this way, a height of MNs can be varied, and a sharp tip radii can be obtained using this low-cost production method [97].

#### 4.2.4. Micromolding Method (Solvent Casting)

**Figure 6:**
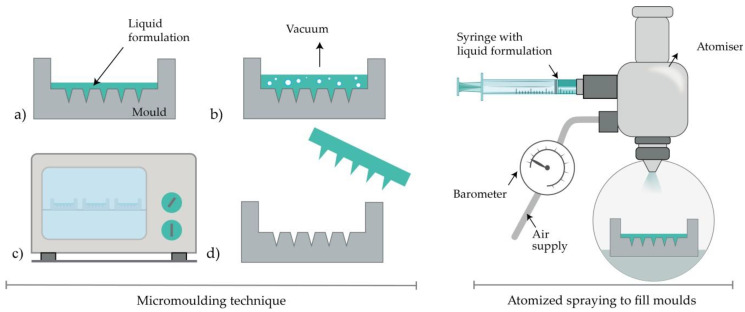
Left: MN production with micromolding (left) consisted of (**a**) pouring the liquid formulation, (**b**) vacuum degasification, (**c**) drying and (**d**) removal of MNs from the mold. Right: Atomized spraying to fill molds.

Dissolving MNs are usually produced by filling a previously prepared MN mold with the liquid formulation [89]. Generally, the mold is made from a silicon wafer as a starting material [10]. Afterwards, the wafer is oxidated at 1000 °C. A needle geometry is patterned using lithography methods, followed by RIE (see Section 4.2.1), while CVD is used for coating a wafer. A liquid polymeric solution is poured into prepared molds, and then, air voids are removed with vacuum or centrifuge [32,106]. Subsequently, the molds are dried in the oven, and MNs are removed after cooling (Figure 6) [110]. The advantages of this method lie in the relatively simple, cost-effective MN production at an ambient temperature [18]. Also, the production of biodegradable polymer MNs, consisting of both natural and synthetic materials, with an appropriate geometry and sufficient strength to penetrate the skin, is reported [10,34]. Interestingly, micromolding has even been used for the production of ceramic MNs [94].

#### 4.2.5. Atomized Spraying Method

This method overcomes the problems associated with the limited capacity for mass production of dissolving MNs with the desired geometry and physical characteristics. Also, the problems linked to the effects of liquid surface tension and viscosity when filling the MN molds can be minimized. Dissolving MN can be produced from the sugars (trehalose, fructose, and raffinose) or polymers (PVA, PVP, CMC, HPMC, and sodium alginate). Briefly, a nozzle connected to an air source and liquid formulation produces an atomized spray (Figure 6). The formulation is filled in PDMS molds and dried for 2h at ambient temperature. Laminate-layered and horizontally-layered dissolving MN can also be produced by this method [101].

#### 4.2.6. Droplet-Born Air Blowing Method (DAB)

Conventional MN production methods have led to drug inactivity due to manufacturing under UV light and heat. The DAB method, proposed by Kim et al., is one of the drawing lithography methods [102]. This method, in which polymer droplets are shaped into MNs with the use of air blowing, enables production under mild conditions, without the use of UV radiation or heat [10].

**Figure 7:**
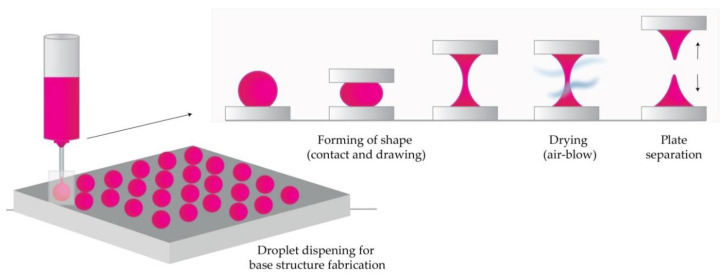
The principle of droplet-born air blowing (DAB) methods (Modified from [123]).

In short, the process begins by dispensing the prepared solution on two plates (upper and lower), then placing the upper plate downwards to allow contact of droplets. The upward movement of the upper plate elongates the viscous solution. In the next step, air blowing removes the residual water and solidifies the droplets in the desired shape by pulling the droplet from a substrate, as illustrated in Figure 7 [102,109,122,123].

The application of one drop of polymer per MN, therefore, enables direct control over the size of drops and the concentration of API. This 10-minute process was used to produce insulin-loaded dissolving MNs that successfully lowered blood glucose levels in diabetic mice [109].

A novel method that uses a shadow mask enabled a uniform MN production, and it overcame low throughput-associated problems in the droplet formation. Using this method, the authors reported controlled drug dosage with optimization of hole width and thickness of the shadow mask [102].

#### 4.2.7. Pulling Pipettes

This method is suitable only for hollow glass MNs. Two research teams produced glass MNs by pulling fire-polished borosilicate glass pipettes exposed at a high temperature with a micropipette puller and beveler [44,86]. Hollow MNs provided an effective delivery of bolus insulin to patients with type 1 diabetes [44]. Overall, it was concluded that glass MNs could infuse millilitres of fluid into the skin [86]. MN produced by this method successfully delivered 6-aminoquinolone and Rose Bengal to the eye, thus enabling intraocular drug delivery in a less invasive and less painful manner than macroscale hypodermic needles [87].

### 4.3. Additive Manufacturing (AM)

Additive manufacturing, more commonly known as 3D printing, represents a new field of research for the manufacturing of MN arrays and molds. The first step in all AM technologies is the design of a 3D object with computer-aided design software (CAD). In the second step, the CAD model is converted to an STL file to tessellate the 3D shape and slice it into digital layers. The STL file is then transferred to the printer using custom machine software, and the printer is set-up with printing parameters. The printer builds the model by fusing or depositing proper material (e.g., ceramics, liquids, thermoplastic, plastic, photopolymer, powders, or even living cells) in layers [124,125,126,127,128,129,130].

Additive manufacturing technologies, FDM, [103,104], photopolymerization-based techniques such as SLA [47,112,113,131,132,133,134], DLP [135,136,137,138] and 2PP [100,114,139,140] were successfully employed in the fabrication of MN arrays. These cutting-edge technologies have numerous advantages over traditional manufacturing approaches including simplicity, low cost, the ability to fabricate complex geometrical products including changes to the original designs at any time, and the production of patient-specific devices [129,141].

#### 4.3.1. Fused Deposition Modelling (FDM)

**Figure 8:**
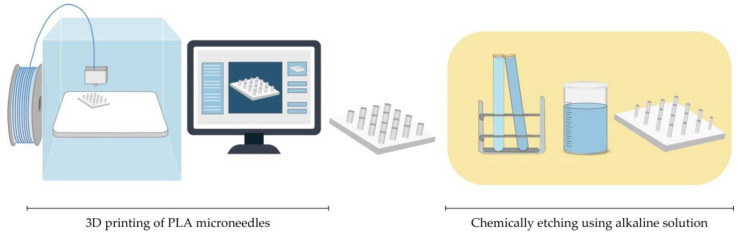
Fabrication of MNs by Fused deposition modelling (FDM) methods, followed by etching in alkaline solution [103,104].

The preparation for printing MNs with typical FDM printers starts with designing MNs using CAD software and optimizing its geometry according to the printer specification [126,130]. Then, the suitable thermoplastic material, in the form of a filament, is fed into the printer by rollers, where it is heated to just above its softening point (glass transition temperature T_g_) by heating elements into a molten state. The melted or softened material, guided by gears, is moved towards the head end where it is extruded from the printer’s head, through a nozzle and subsequently deposited layer-by-layer on a build plate, cooling and solidifying in under a second (Figure 8) [124,125,126,127]. The printer’s head moves within the x- and y-axes, whereas the platform can move within the z-axis, thus creating 3D structures [129].

Standard filaments used in FDM printers are acrylonitrile butadiene styrene (ABS), PLA, PVA, high impact polystyrene (HIPS), polyethylene terephthalate glycol-modified (PET-G), and nylon, while the dimensions of filaments adopted in the commercially available print head are in the range of 1.75 mm and 2.85–3 mm [126].

Processing parameters that should be optimized during an FDM process include nozzle diameter, feed rate, the temperature of both the nozzle and the building plate, printing speed, the height of the layers, and part built orientation [127,129].

Although FDM is a versatile and cost-effective MNs manufacturing method, its main limitation is low printing resolution. Luzuriaga et al. reported for the first time combination of FDM with a post-fabrication etching step to obtain ideally sized and shaped needles [104]. Camović et al. also successfully used FDM to print MNs, which were subsequently coated [103] (Figure 8).

#### 4.3.2. Stereolithography (SLA)

With its high resolution and accuracy, as well as smooth surface finish, SLA is the most commonly used technology for printing MNs. Ovsianikov et al. were the first to report that the lithography-based multiphoton polymerization 3D printing method can be used to create MN arrays for transdermal drug delivery [73]. This method is based on the photopolymerization of liquid resin with photo-active monomers by UV light. MNs are built by solidification of subsequent layers of resin in the presence of high energy light, e.g., UV laser beam guided by scanner mirrors [126]. MN pattern is created by a laser beam on the surface of a resin, which causes the resin to have definite depth. To remove unpolymerized resin residues, MNs are washed in an alcohol bath and then cured in the UV chamber [113,133].

Although SLA prints high-quality parts at a fine resolution (range of 10 μm), this method is relatively slow, expensive, and the range of printing materials is very limited (lack of biocompatibility) [142]. Many research groups reported using this photopolymerization-based technique in MNs manufacturing, to obtain solid MNs, hollow MNs [47], and MN molds [131]. Pere et al. and Economidou et al. employed SLA to fabricate MN arrays using a Class 1 biocompatible resin, with excellent mechanical strength, which was coated with insulin–sugar films [113,133].

#### 4.3.3. Digital Light Processing (DLP)

DLP is also a photopolymerization-based technology based on the ability to polymerize photosensitive polymers through projections of light. This method is faster than SLA, and a high definition projector flashes the entire cross-section of the object at once, in the form of volumetric pixels [124]. Gittard et al. reported that DLP could be used in MNs fabrication. In their study, they successfully employed DLP to print solid MN array structures in various geometries out of an acrylate-based polymer for wound healing applications [135]. El-Sayed et al. also successfully used a desktop DLP 3D printer for MNs molds for nanoparticle delivery [137]. Lu et al. fabricated drug-loaded MN arrays for the transdermal delivery of a chemotherapeutic drug using microstereolithographic (DLP) apparatus.

#### 4.3.4. Two-Photon-Polymerization (2PP)

2PP method enables the low-cost, layer-by-layer fabrication process of 3D structures from solid, liquid, or powder precursors in the microscale and nanoscale structures. A femtosecond or picosecond laser is focused inside a liquid resin droplet for the polymerization into MN structure [95,124]. The process is based on the temporal and spatial overlap of photons to achieve photopolymerization [100]. The advantages of the process encompass a high level of flexibility, scalable resolution, improved geometry control, and also, the process can be performed in conventional facilities [95,124].

Doraiswamy et al. first reported using 2PP to produce MNs from Ormocer^®^ (organically modified ceramic) materials [85]. Trautmann et al. reported using 2PP to fabricate hollow MNs combined with internal laser-generated microchannels [139]. Another research group also printed ultra-sharp polymer MNs via 2PP [140].

Cordeiro et al. described an approach to fabricate high-quality MN array master templates using 2PP 3D [114]. Gittard et al. suggested that 2PP can create MNs with a wide range of geometries (in-plane, out-of-plane, rocket-shaped, and mosquito fascicle-shaped MNs) [100].

### 4.4. Microneedle Coating Techniques

Solid MNs can be coated with the drug-containing dispersion, which provides a rapid drug release from the coating into the tissue [143]. Depending on the way that the physical contact between MN and drug-containing dispersion is achieved, some methods imply the selective coating of the MN shafts only while other methods include the coating of both the shafts and the base of substrate. Minimal drug loss, better control over drug dosage, and efficient delivery can be accomplished if the MNs are selectively coated. Good quality of coating, reproducible coating process, and efficient delivery are three factors that play a main role in providing versatile application of MNs [144].

However, it is hard to achieve a suitable drug release profile because of the limited surface where the drug can be inserted, which is caused by specific MN’s structure. Problems with stability, uniformity, consistency, and reproducibility may also occur. During the coating process, the drug can be lost from the MN surface. The non-uniform coating thickness of the drug on the MN surface may lead to inaccurate dosing. All those drawbacks should be considered when choosing the right method for coating MNs with drug-containing dispersion [143].

#### 4.4.1. Dip-Coating

Dip-coating is a method that selectively coats the MN shaft without contaminating the base substrate of the MN array by immersing MNs in drug formulation. Different aqueous, organic solvent-based, or molten liquid can be used. Dipping results in forming a liquid film on the MN surface followed by drying where the adherent liquid film is converted into a solid coating [119,143]. The viscosity and surface tension of the coating solution should be adjusted carefully to prevent the rising of coating solution upon the MN shaft to the base substrate. The selective coating of the MN shaft can be achieved by masked dip-coating or thin-film dip-coating. Masked dip-coating implies the use of a masking plate, which disables the passing of the coating solution to the base substrate [145]. In thin-film dip-coating, the thickness of the coating solution is lower than the height of the MN, which assures the insignificant capillary rise of the coating solution and, therefore, prevents possible contact between coating solution and base substrate [119].

The drug amount that is coated on the MNs is dependent directly on the thickness of the coating on the MN shaft. The higher thickness and drug mass can be achieved by raising the speed at which MNs exit the coating solution, enhancing solution viscosity and increasing the number of dipping. Adding surfactants ensures uniform and integrated coating and decreases the surface tension of the solution. Drying time between dips also impacts the coating thickness [144].

Simple fabrication process and low costs make dip-coating a very convenient method for MN fabrication. Optimal drug delivery can be achieved by upgrading the method with a dam board, a roller, a fixture, and a limit [146]. The main drawback of this method is slow drying that can cause loss of drug dispersion from the surface of MNs. Also, surface tension can obstruct the uniform coating of the individual MNs if they’re closely spaced [119].

Solid MNs were fabricated from stainless steel using laser cutting and electropolishing. Then, suitable concentrations of CMC and Lutrol F-68 NF were used to increase viscosity, decrease the surface tension of the coating solution, and avoid contamination of the base, with micron-scale control over the length of the coated shaft. The MNs were coated with vitamin B, calcein, bovine serum albumin, plasmid DNA, and viruses [22]. Titanium MNs, dip-coated with recombinant human growth hormone, provided similar absolute bioavailability as commercial subcutaneous injections. The authors concluded that the lack of pain and ease of administration might lead to the replacement of these injections with MN patches [99].

MNs coated with vaccines should be targeted to skin immune cells where the preservation of protein integrity is important because a change in protein structure can lead to impaired vaccination efficacy and an altered immune response [46]. MN bases were coated with an assembly of DNA vaccines, pH-responsive copolymer, and heparin. The release of vaccines was enabled by electrostatic repulsion between co-polymer and heparin [147]. DNA vaccines have an intracellular encoded antigen, which is presented directly to essential effector cells for cytolytic activity. MNs provide penetration through the epidermis of the skin to deliver DNA vaccines to the resident antigen-presenting cells within the dermis. They also enable intracellular co-delivery of DNA vaccines by using polyelectrolyte multilayers with adjuvant materials [148]. The main disadvantage of MNs coated with DNA vaccines is poor coating efficiency and immunogenicity. Nano-patterned MNs improved the affinity of stainless steel for plasmid DNA and consequently enhanced vaccine efficiency and its immune response. Nano-patterned MNs had better dip-coating efficiency and DNA vaccine loading capacity because of their more hydrophilic surface. Better cytocompatibility was accomplished according to higher cell proliferation. Most importantly, they had a higher level of cellular immune responses [149].

Antigen activity can be decreased when influenza vaccine is coated on MNs. CMC increased viscosity but also contributed to vaccine activity loss due to virus particles aggregation. Replacing CMC with trehalose assured protection of the antigen and its activity due to blocked particle aggregation and better thermal stability [150].

Layer-by-layer coating represents a modified dip-coating method. Electrostatic interactions are used to create a layered coating on the MN surface, unlike the classic coating method where the coating is based on the viscosity of the solution. In the case of DNA or protein molecules, the solution contains negatively charged DNA and positively charged polymer, which leads to the formation of a polyelectrolyte multilayer on the MNs. Chemically modifying the MN surface or precoating multiple alternate layers of negatively and positively charged polymers is necessary to acquire the desired charge polarity [151].

Drop coating is another modification of the dip-coating method. It implies dropping the coating solution on the MN array instead of dipping the MN array into the solution. Slow solvent evaporation leads to a non-uniform coating of the MNs and the base, liquid segregation from the MN tip, and substrate accumulation between MNs. It leads to the stage where the mostly coated area is the base. These drawbacks can be exceeded by heating the patch or drying under vacuum [152].

#### 4.4.2. Gas-Jet Drying

**Figure 9:**
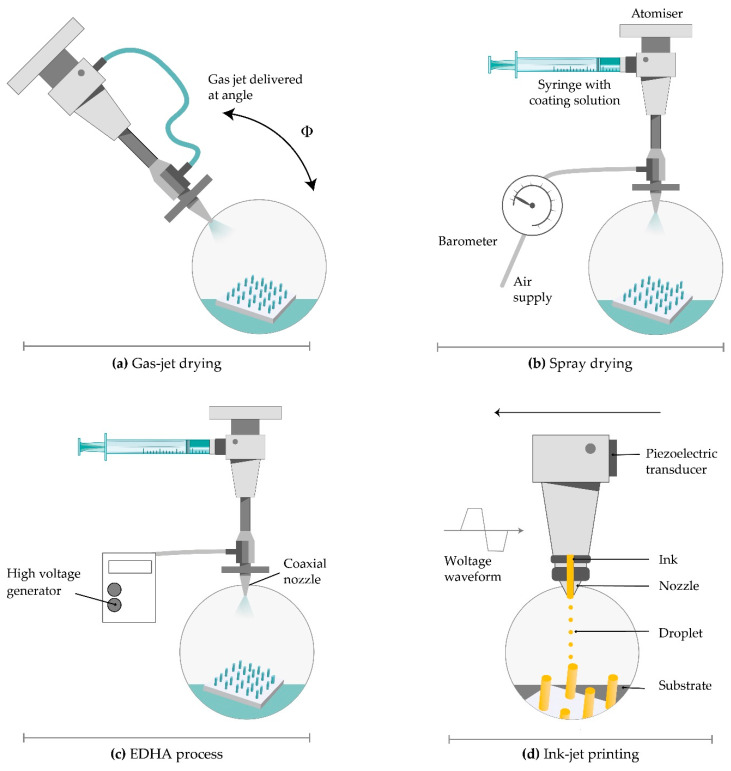
Coating techniques for MNs. (**a**) gas-jet drying; (**b**) spray drying; (**c**) electrohydrodynamic atomization (EHDA) processes; (**d**) ink-jet printing.

Gas-jet drying is a method where the drug suspended in a coating solution gets transitioned into the gas phase using a gas-jet applicator (Figure 9) [153]. It is suitable especially for curved MNs, because the slow drying process, specific for dip-coating, is not convenient in this case. Wet coating liquid on the surface of the MNs has the potential to move and change its thickness and, consequently, the dose accuracy. This method is also appropriate for small (<90-micron length) and very closely spaced (~20,000 cm^−2^) MNs [154].

Solid silicon microprocjections were coated with a thin layer of gold. The solution, with ideal surface tension and viscosity, including methylcellulose, surfactant and model drug, is coated on the whole length of the microprojection. Drying started with a gas jet at 6–8 m/s and incident angle of 20° horizontally to direct the coating liquid onto the MNs and away from the base. The thickness of the coated layer on microprojections was 5 µm and increased rapidly to allow the coated material to dry instead of relocating on the base substrate. This method offers several advantages including uniform distribution and fast drying of the coating solution, more or less constant viscosity of the bottom layer of the solution, and the possibility of removing the excess coating solution from the base substrate [155]. 

The improved delivery of large vaccine molecules, through the SC, can be achieved by modifying the gas jet method for coating MNs. Raising the incident angle from 20° to 70°, removing the patch edge, and rotating the patches during the coating process ensured the uniformity and relocation of the drug from the whole MNs only to the tips. Much lower doses provided an equivalent protective immune response as the intramuscular injection. These MN patches also contributed to extended and improved vaccine stability [156].

#### 4.4.3. Spray Coating

Spray coating implies using fluid pressure to create droplets. An intact film-coat is formed from fine droplets (<280 µm), which are deposited on MN array and then, outspread and coalesced. The first step is atomization, which generates fine droplets (Figure 9). Then comes the deposition and adherence of droplets, which collide on the surface. The last step is a coalescence of droplets on the substrate to form an intact film coating [64]. 

The nozzle design, concentration, input ray, physicochemical characteristics of the coating solution (viscosity, surface tension, and density), and processing parameters like air-to-liquid mass ratio, the duration of spraying, atomization air pressure, gun-to-surface distance, and air cap setting determine the droplet size. The deposition of droplets on the surface is determined by spray velocity and spray density [64]. The spray coating process can be used for the efficient application of an intact, micron-sized film-coating on silicon MN arrays as well as the production of dissolved MNs (see Section 4.2.5). 

McGrath et al. used a nozzle, connected to a compressed air source and coating solution, for the production of an atomized spray. They fixed silicon MN patches to the adjustable stage by tape and used a syringe driver and peristaltic pump to control the rate of liquid input. A coating solution made of HPMC, CMC, and surfactant provided fast film-forming and enhancement of the coalescence of droplets on the MN surface [64].

#### 4.4.4. Electrohydrodynamic Atomization (EDHA)

Electrohydrodynamic atomization (EHDA) is a production method of atomized droplets by a moving liquid where charge inside the droplets is generated by the electrical field. When the critical voltage is achieved, liquid spurts out of a nozzle in the form of droplets. Then, it is deposited onto a grounded collector positioned below the nozzle tip (Figure 9) [157]. The coating liquid contains a solvent, polymer, and drug. EHDA can generate particles (electrospraying) and fibres (electrospinning). This method provides coating the MN tips only, without coating the base substrate due to insulating polymeric masks. Still, there is a lot of drug wastage on the mask upon the base substrate [158].

The EHDA process can be single needled (formulation is injected into a single nozzle by syringe pump), coaxial (two or more immiscible liquids are put in separate nozzles), and multiplexed (formulation is put in a single or coaxial nozzle array). The coaxial system protects the drug from direct exposure to the environment and enables sustained and controlled drug release [154]. Flow rate, voltage, and distance between the nozzle and collecting platform and solution viscosity and surface tension have a significant influence on particle size, size distribution, porosity, shape, and surface charge. The material characteristics affect jet stability. The most important requirement for the EHDA process is a low electrical conductivity of the solvent [158].

This method is used for the delivery of insulin, folic acid, titanium dioxide antimicrobial agent, gold used in gene delivery, and sensitive biomolecules like peptides and proteins, which are unstable when administrated orally [158,159]. Ali et al. coated MNs with particles and fibres through the EHDA process. They concluded that MNs with PVP in ethanol showed more rapid release compared to a sustained release profile of MN coated with polycaprolactone in dichloromethane. Both types of MN successfully penetrated through the skin, and the electrospun MN coating released a large amount of the loaded drug within 6 h [160]. 

Angkawinitwong et al. used the EHDA process to coat ovalbumin loaded-PLGA nanoparticles onto hydrogel-forming MN arrays. The extended-release of ovalbumin over 28 days was observed. Uniform particle coating on the MNs was perceived by microscopic analysis. The mechanical and insertion properties of the coated MNs were the same as the uncoated MN, suggesting that the coating did not harm the application of MNs [161].

#### 4.4.5. Piezoelectric Inkjet Printing

Piezoelectric inkjet printing provides a controlled and precise MN coating with liquid droplets (1–100 pl), which is followed by solidification. The method is compatible with different aqueous and organic solvents. The low viscosity of the formulation is preferable to prevent clogging the small jetting nozzle. The voltage supplied material connected to a piezoelectric transducer produce vibrations to eject drops from the nozzle [162].

A modified method called thermal inkjet printing implies drop production by increasing the formulation temperature a bit higher than its boiling point (Figure 9) [162]. The pressure pulse in the ink chamber made by an electric field distorts piezoelectric crystal and force drops ejection from the nozzle. The nozzle dimensions determine the droplet size. The coating deposition is mainly dependent on nozzle size, applied voltage, and pulse duration [163]. The variation of this continuous inkjet printing is dropped on-demand printing. It implies liquid ejection from a printhead only when a drop is required [164]. 

This method provides coating MNs with poorly soluble drugs. Biodegradable PGA MNs were coated with voriconazole and showed antifungal activity [165]. The transdermal delivery of 5-fluorouracil, curcumin, and cisplatin can be achieved by metallic MN produced by piezoelectric inkjet printing. Drug solubility directly affected the release profile and concentration affected antiproliferative activity [163]. Although piezoelectric inkjet printing has advantages regarding dip-coating, such as improved accuracy, reproducibility, reduced waste, scalability, and its amenability for continuous manufacturing, this method is limited by the available MN surface area that can be directly targeted for printing [166].

### 4.5. From Clinical Trials to Commercial Development

Up to now, MNs with a broad range of geometries, with or without a MN application device, have been fabricated using different manufacturing methods from a variety of materials. Although MNs have been extensively studied for transdermal drug delivery and vaccine delivery, these systems can also be designed for delivery targeted to other tissues such as oral mucosa, vaginal mucosa, anal sphincter muscles, and hair follicles [167]. Nowadays MNs are also being explored for ocular drug delivery where the drug is delivered to the cornea, sclera, and suprachoroidal space [168]. MNs are already in advanced development and marketed for cosmetic skincare (Dermaroller^®^, Dermapen^®^). Recently, MNs were investigated as a part of the monitoring/diagnosis system to provide the ability for at transdermal sampling of body fluids in a completely painless manner [169]. During the last 5 years, there have been a variety of completed clinical trials involving the use of MNs as can be seen in Table 2. The majority of these clinical trials involve the use of MN injection systems and MN array-based patches, in order to prove the efficacy and safety of MN delivery systems versus traditional delivery systems.

It is necessary to adequately select the type of MNs and their geometry in order to successfully developed the final product with sufficient and reproducible penetration for transdermal drug delivery. Usually, in order to obtain the possibility of self-administration a MN application device (manual hand-held or impact-insertion) is necessary. Many pharmaceutical companies and research laboratories are involved in the MN-based product development including 3M, Zosano Pharma, Alza Corporation, Becton-Dickinson Technologies, Valeritas, Vaxxas, Microneedle Therapy System, Nanopass Technologies Lohmann, Therapie- Systeme AG, and others.

**Figure 10:**
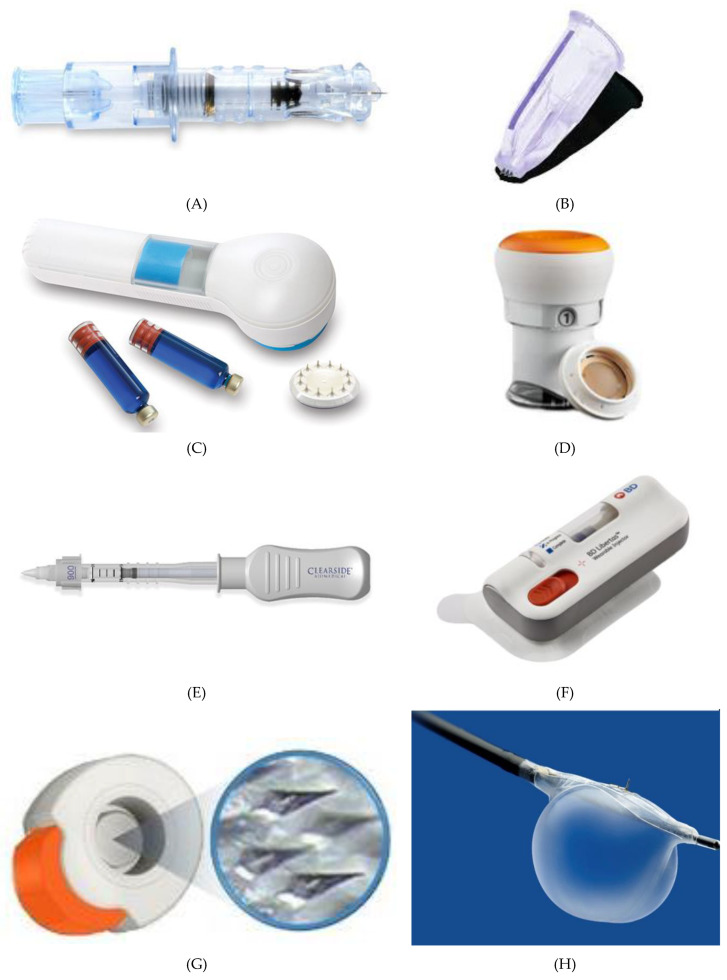
Current MN devices. (**A**) Soluvia^®^, (**B**) MicronJet^®^600, (**C**) Microstructured Transdermal System^®^, (**D**) Qtrypta^TM^, (**E**) SCS Microinjector^®^, (**F**) Microinfusor^®^, (**G**) MicroCor^®^, (**H**) Bullfrog^®^ Micro-Infusion Device.

Most of the MN devices are still in clinical trials, and only a few of them are currently available in the market. The first commercialized MN device was developed by Becton-Dickinson Technologies named Soluvia^®^ (Figure 10A) although some authors suggest that this device does not contain truly MN arrays, but rather very short hollow needles that allow successful ID injection from a conventional syringe barrel [18]. Sanofi Pasteur marketed Intanza^®^ in 2009 as the first influenza vaccine that targets the dermis [170]. Even though many clinical studies indicated that Intanza’s benefits are greater than its risks, in 2018 the product is withdrawn from use in the European Union at the request of the marketing authorization holder [171]. In February 2010, the FDA approved MicronJet^®^ by Nanopass Technologies. This single-use MN device composed of four hollow silicon needles shorter than 500 mm in length attached to a plastic device, was used to deliver insulin, lidocaine, and influenza vaccine intradermally. In 2009, the company completed Phase 1 clinical trial with the aim of comparing glucose pharmacokinetics and insulin pharmacodynamics injected via the Micronjet^®^ with a conventional needle for the delivery of insulin [172]. To improve device performance, especially the insertion technique, the company developed MicronJet600^®^ as a new device version [173]. In 2019, Yonsei University completed a clinical study to evaluate the safety and immunogenicity of Bacillus Calmette–Guerin (BCG) delivery via Micronjet600^®^ device (Figure 10B) compared to those via a conventional needle [174].

3M^^TM^^ developed the Microstructured Transdermal System (MTS) (Figure 10C) including hollow MN (hMTS) and solid MN technology (sMTS). This system consists of a coated MNs which allow water-soluble, polar, and ionic molecules, such as lidocaine, to be successfully delivered through the skin within seconds. Hollow MTS is now available for use in clinical trials, while sMTS was successfully used in Phase I and II clinical studies [175]. In 2019, Radius Health, Inc. started a phase III clinical study on the delivery of abaloparatide in the treatment of postmenopausal women with osteoporosis by using sMTS [176].

Zosano Pharma Corporation introduced their Zosano patch-coated titanium microprojections array designed for the enhanced delivery of biopharmaceuticals such as protein, peptide, vaccines, and other biologics into the skin. Recently, Zosano Pharma Corporation developed Qtrypta^TM^ (Figure 10D) (zolmitriptan intracutaneous microneedle system) for the acute treatment of migraine with or without aura in adult patients, which is currently under review by the FDA (will be available, if approved, in 2021) [177,178].

The SCS Microinjector^®^ (Figure 10E) is Clearside Biomedical proprietary, which is composed of a syringe and two 30-gauge hollow MNs of varying lengths, each less than 1.2 millimetres used to inject a wide variety of drugs into the suprachoroidal space [179].

The Microinfusor^®^ (Figure 10F), developed by Becton Dickinson (BD) Technologies, is a hollow MN system that allows delivery of a wide range of drugs to the subcutaneous tissue over a period of time. Corium’s MicroCor^®^ system (Figure 10G) consists of dissolving MNs for an innovative, needle-free system delivery of drugs and vaccines across the skin. The company successfully demonstrated the ability to incorporate a wide range of molecules into the MicroCor^®^ system, although the safety and efficacy of MicroCor^®^-based products have not yet been established [180].

Mercator MedSystems, Inc. developed a very interesting MN-based device named Bullfrog^®^ Micro-Infusion Device (Figure 10H) in order to safely inject therapeutic molecules through blood vessel walls into adventitial tissues. The device is tipped with a balloon-sheathed MN. This device has received 510(k) marketing clearance from the FDA is CE Marked [181].

Above mentioned MNs devices, especially marketed ones, should encourage researchers and companies to move toward large-scale manufacture and design of MN devices using different novel materials and production methods.

## 5. Conclusions

Although TDDs were initially limited to several APIs with suitable physicochemical properties for passage through the SC, MNs have emerged as promising DDS for transdermal drug delivery. MNs offer prospects for the development of personalized DDSs, which should lead to better patient compliance and adherence due to their ease of handling by patients at home. As this field continues to evolve, MN-based devices that are inexpensive, smaller, reusable, and pain-free are being developed.

With the advancement of MN technologies, manufacturing methods for MNs are becoming more sophisticated. MNs can, therefore, even be integrated into lab-on-a-chip devices and some of the point-of-care devices, providing the potential for both diagnostic and therapeutic applications. However, selecting the optimal material, MN geometry and manufacturing process is and remains crucial for optimum results.

A large number of clinical trials on MNs demonstrate the worldwide interest of the scientific community in the use of devices in various therapeutic indications. Therefore, some of the MN devices have reached the commercial market. MN-based (trans)dermal drug delivery could play a significant role in the modern healthcare system in the future and the success of these minimally-invasive devices would also open up a wide range of therapeutic opportunities for buccal, oral, vaginal, rectal, and ocular drug delivery.

## Figures and Tables

**Table 1:** Suitable materials for microneedle (MN) manufacturing.

Material Type		MN Type	Manufacturing Method	Reference
α- aluminium (III) oxide (α-Al_2_O_3_), zirconia		Ceramic (solid), hollow	Lithography and ceramic sintering, micromolding, two-photon polymerization (2PP)	[73,85]
Glass		Hollow	Pulling pipettes	[44,86,87]
Mesoporous silicon		Coated	Post-synthesis grafting method	[88]
Nickel/iron		Solid, hollow, coated	Laser-ablatedion, micromolding, electroless plating, wet etching	[89,90]
Nitinol		Hollow	Multiple-pulse laser microhole drilling	[74]
Silicon		Solid, hollow, coated	Etching, lithography	[30,35,91,92]
Stainless steel		Solid, hollow, coated	Laser cutting, laser ablation, etching, electroplating, electropolishing, lithography, and microstereolithography	[22,25,51,71,72,93,94,95,96,97]
Titanium		Solid, hollow, coated	Microelectromechanical systems (MEMS)	[98,99]
Natural polymer	Amylopectin	Dissolving	Photolithography	[67]
	Chondroitin sulphate	Hollow	2PP	[100]
	CMC	Hollow, dissolving	2PP, droplet-born air blowing (DAB) method	[100,101,102]
	Dextran	Hollow	2PP, atomized spraying process	[100]
	Galactose, trehalose, maltose fructose, raffinose	Solid, dissolving	Micromolding, atomized spraying process	[31,80,81,82,83,84,101]
Biodegradable synthetic polymer	Thermoplastic starch	Dissolving	Electro-discharge machining process	[63]
	PLA	Solid, dissolving	Fused deposition modelling (FDM), micromolding	[10,103,104,105]
	PLGA	Hollow, solid, dissolving	2PP, micromolding	[10,100]
	Polycarbonate	Solid	UV lithography, electroforming	[14]
	PMVE/MA copolymer	Polymeric, hydrogel	Laser-based method for micromolding, micromolding	[32,106]
	PVA	Dissolving, hydrogel	Atomized spraying process	[101]
	PVP	Dissolving, hollow	2PP, atomized spraying process	[100,101]

**Table 2:** MNs in completed clinical trials (2015–2020).

Title of the Study	Aim of the Study	Condition	Phase	Type/Material of MNs	Device	Year
The effect of microneedle pretreatment on topical anaesthesia	Evaluation of the role of MN pretreatment in the speed at which anaesthesia develops after application of topical 4% lidocaine	Pain	-	Solid/Metal (stainless steel)	MN Roller	2015
Safety demonstration of microneedle insertion	Observation of biocompatibility and inertness of gold- or silver-coated, or uncoated nickel MNs	Allergic reaction to nickel	-	Solid/Metal (gold- or silver-coated, or uncoated nickel MNs)	MN patch	2015
Safety study of suprachoroidal triamcinolone acetonide via microneedle to treat uveitis	Evaluation of the safety, tolerability, and procedure of a MN injection of triamcinolone acetonide into the suprachoroidal space	Uveitis	Phase 1 Phase 2	Hollow	Single MN (SCS Microinjector^®^)	2015
Safety and efficacy of ZP-glucagon to injectable glucagon for hypoglycemia	Comparison of Zosano Pharma Glucagon transdermal patch system and conventional glucagon injection	Hypoglycemia	Phase 1	Solid/Metal (drug-coated titanium MNs)	Zosano MN patch	2015
The use of microneedles to expedite treatment time in photodynamic therapy	Investigation of varying incubation periods of topical aminolevulinic acid after pretreatment with MN application in photodynamic therapy	Keratosis, actinic	-	Solid/Metal (stainless steel)	MN Roller	2016
Clinical evaluation of healthy subjects receiving intradermal saline using the microneedle adapter (Model UAR-2S)	Evaluation of the MN Adapter performance in healthy subjects in 3 different injection sites	Intradermal injection	-	Solid/Metal (gold-coated metallic MN)	Microdermics Inc. MN Adapter (model UAR-2S)	2017
Safety and efficacy of ZP-zolmitriptan intracutaneous microneedle systems for the acute treatment of migraine (Zotrip)	Comparison of safety and efficacy of a range of doses of Zolmitriptan intracutaneous MN systems and placebo	Acute migraine	Phase 2 Phase 3	Solid/Metal (drug-coated titanium MNs)	Adhesive dermally applied microarray (ADAM) by Zosano	2017
Glucose measurement using microneedle patches	Comparison of a MN patch versus a lancet or intravenous catheter, in monitoring glucose levels	Diabetes	-	Solid/Metal or biocompatible polymers	MN patch	2018
A study to evaluate the long-term safety of M207 in the acute treatment of migraine (ADAM)	A Long-term, open-label study to evaluate the safety of M207 (zolmitriptan intracutaneous MN system) in the acute treatment of migraine	Migraine	Phase 3	Solid/Metal (drug-coated titanium MNs)	Adhesive dermally applied microarray by Zosano	2019
Microneedle patch study in healthy infants/young children	Evaluation of safety, reactogenicity, and acceptability of placebo MN patch placement to the skin of children	Vaccination. skin absorption	–	Solid/ Water-soluble excipients	Single patch	2019
Proof-of-concept study of LymphMonitor 1.0 to assess the lymphatic vessel function	Testing how efficiently the lymphatic system is functioning using LymphMonitor 1.0 (a solution of a fluorescent dye, indocyanine green)	Lymphedema	Phase 1	Hollow/ Silicon MNs	MicronJet600^®^	2020

Data obtained from https://clinicaltrials.gov.
